# Apigenin Alleviates Zearalenone-Induced Oxidative Stress and Apoptosis in Swine Testis Cells Through the Wnt Signaling Pathway

**DOI:** 10.3390/antiox15010042

**Published:** 2025-12-29

**Authors:** Chenyun Guo, Yidan Zhang, Yiwei Wang, Yile Sun, Haoze Ning, Jiaxin Gao, Fei Guo, Pengyun Ji, Lu Zhang, Guoshi Liu, Bingyuan Wang

**Affiliations:** 1 College of Animal Science Technology, China Agricultural University, Beijing 100193, China; s20243040827@cau.edu.cn (C.G.); zhangyidan112@cau.edu.cn (Y.Z.); s20233040771@cau.edu.cn (Y.W.); sy20243040989@cau.edu.cn (H.N.); sy20243040905@cau.edu.cn (J.G.); guofei@cau.edu.cn (F.G.); jipengyun@cau.edu.cn (P.J.); luzhang2018@cau.edu.cn (L.Z.); 2 College of Animal Science and Technology, Heilongjiang Bayi Agricultural University, Daqing 163711, China; 120240160@taru.edu.cn; 3 Institute of Animal Sciences, Chinese Academy of Agricultural Sciences, Beijing 100193, China

**Keywords:** Swine Testis cells, zearalenone, apigenin, apoptosis, LRP5, oxidative stress

## Abstract

The mycotoxin zearalenone (ZEN), commonly found in contaminated food and feed, poses a significant threat to human and animal health, particularly to reproductive function. Mitigating its toxicity represents a critical research priority in food safety. Apigenin (AP) is a naturally occurring dietary flavonoid with phytoestrogenic properties and exhibits diverse pharmacological activities. In this study, we investigated the protective effects of AP against ZEN-induced apoptosis and oxidative stress in Swine Testis (ST) cells and elucidated its underlying mechanisms. The identity of ST cells was verified via RT-PCR and agarose gel electrophoresis. ST cells were treated with 40 μM ZEN and 1 μM and 0.1 μM AP for 24 h. Cell viability was detected via CCK8 and EdU assays, cytotoxicity was evaluated via LDH assay, cell cycle and apoptosis levels were analyzed via flow cytometry, and the mechanism by which AP alleviated the damage caused by ZEN to ST cells was preliminarily revealed using RNA-Seq technology. Further, the expression levels of related genes and proteins were detected by qRT-PCR and Western blot. Our results show that 1 μM or 0.1 μM AP effectively attenuated the cytotoxicity induced by 40 μM ZEN in ST cells, as evidenced by restored cell viability, reduced the LDH level, normalized cell cycle progression, reduced apoptotic rates, and enhanced antioxidant capacity. RNA-Seq analysis was coupled with molecular validation and used to elucidate the mechanisms underlying AP-mediated protection against ZEN-induced cellular injury. It is shown that ZEN suppressed the expression of LRP5, a pivotal gene in the Wnt signaling pathway, along with its downstream effector c-Myc. Conversely, treatment with 1 μM or 0.1 μM AP upregulated the expression of LRP5, iASPP, and TRAF2 at both transcriptional and translational levels. Importantly, the protective effects of AP were abrogated with IWR-1-endo, a specific Wnt pathway inhibitor, confirming pathway dependency. Collectively, our findings show that AP alleviates ZEN-induced oxidative stress and apoptosis in ST cells through the upregulation of LRP5 and subsequent activation of the Wnt signaling pathway. This study provides molecular evidence supporting the potential clinical application of AP as a preventive agent against ZEN-induced reproductive toxicity.

## 1. Introduction

Zearalenone (ZEN), also known as F-2 toxin, is a non-steroidal estrogenic mycotoxin predominantly produced by Fusarium species and represents one of the three major mycotoxins found in animal feed [1]. ZEN is ubiquitously present in cereal grains, including corn, barley, wheat, sorghum, and oats [2], as well as their processed by-products, which constitute essential components of both human and animal diets. Under standard conditions, ZEN manifests as white crystalline particles and exhibits remarkable thermal stability, demonstrating resistance to degradation during food processing and storage [3]. Following the ingestion of contaminated feed or food, ZEN can induce multiple adverse effects in both humans and animals. Unlike other mycotoxins, ZEN possesses a structural similarity to endogenous estrogen, enabling it to disrupt normal estrogen homeostasis and subsequently leading to reproductive dysfunction [4]. In females, ZEN exposure can trigger aberrant follicular development and ovulatory disorders [5]; in males, it interferes with testosterone secretion, resulting in impaired spermatogenesis, diminished sperm quality, elevated sperm deformity rates [6], and the induction of germ cell apoptosis [7], thereby contributing to various reproductive pathologies [3]. Moreover, ZEN exhibits diverse additional toxic effects, including immunotoxicity [8], hepatotoxicity [9], and nephrotoxicity [10].

Based on the mechanisms delineated above, reproductive toxicity is considered a primary target of ZEN toxicity, with swine demonstrating particularly high susceptibility to its effects. Accumulating evidence has demonstrated that ZEN suppresses the viability of porcine endometrial stromal cells [11], induces apoptosis and oxidative stress in Swine Testis (ST) cells [12], and triggers oxidative stress and autophagy in ST cells of piglets [13]. Furthermore, ZEN-fed bucks have exhibited deteriorated sperm quality, structural damage to seminiferous tubules, and compromised blood–testis barrier integrity [14]. In weaned piglets, ZEN accelerates ovarian development through promoting follicular proliferation [15] and can also induce abnormal uterine hyperplasia [16]. In humans, ZEN disrupts endocrine function by binding to α and β estrogen receptors [17], triggering a cascade of detrimental effects, including impaired development and inflammation/hypertrophy of sexual organs, induced apoptosis necrosis of germ cells and reduced germ cell viability, ultimately leading to diminished reproductive capacity [18,19,20]. Given the substantial damage ZEN inflicts upon the reproductive systems of both humans and animals, developing effective strategies to mitigate its reproductive toxicity and control ZEN contamination in cereal grains has emerged as a critical research priority in food and feed safety.

The testis is a major target organ for male reproductive toxicants. Within its seminiferous tubules, Sertoli cells perform essential functions, including providing nutritional and morphogenetic support to germ cells, thereby underpinning spermatogenesis [21]. During embryonic Sertoli cell lineage differentiation, relevant genes, including WT1, GATA4, SOX9 and AMH, are expressed at specific times and in specific locations to ensure the correct differentiation of the embryo toward the male phenotype [22]. UCHL1, a well-acknowledged marker for porcine immature spermatogonia, and ZBTB16 are crucial to the maintenance and self-renewal of undifferentiated spermatogonia [23]. In addition, Sertoli cells establish the blood–testis barrier (BTB) to provide a protected microenvironment for germ cell development [24]. Consequently, spermatogenesis, which is heavily reliant on Sertoli cells, is highly vulnerable to any toxicant that impairs the viability or function of these cells.

Accumulating evidence suggests that certain antioxidants can effectively ameliorate ZEN-induced toxicity, including procyanidins [12], lactic acid bacteria [25], melatonin [26], and rutin [11]. These findings substantiate the therapeutic potential of antioxidants in counteracting the toxic effects of ZEN. Apigenin (AP, also designated as 4′,5,7-trihydroxyflavone) is a flavonoid compound widely distributed in various fruits and vegetables, including celery, chamomile, onions, honey, and malt [27,28]. As a phytoestrogen [29], AP has been revealed to possess multiple pharmacological properties, encompassing anti-inflammatory, antioxidant, antibacterial, and anticancer activities [30]. Research has indicated that AP promotes cell proliferation and attenuates lipopolysaccharide (LPS)-induced apoptosis [27]. Regarding reproductive protection, AP ameliorates doxorubicin-induced testicular injury [31], alleviates acrylonitrile-induced inflammation and apoptosis in rat testicular cells [32], and exerts protective effects against acrylonitrile-induced subchronic sperm injury by improving sperm concentration and motility [33]. Additionally, AP inhibits LPS-induced mitochondrial membrane depolarization in endothelial cells and restores mitochondrial complex I activity [27]. Notably, the biological functions of AP exhibit dose-dependency and tissue-specific variations [34].

While previous studies have documented the deleterious effects of ZEN on the male reproductive system, it remains unclear whether AP can counteract ZEN-induced reproductive toxicity and the specific molecular mechanisms underlying such protection. Therefore, the present study aims to investigate whether AP exerts protective effects, including proliferation promotion, apoptosis inhibition, and antioxidant capacity enhancement in ST cells following ZEN-induced injury. Furthermore, by employing transcriptome sequencing technology to identify relevant signaling pathways, we sought to elucidate the molecular mechanisms through which AP alleviates ZEN-induced injury in ST cells.

## 2. Materials and Methods

### 2.1. Chemicals and Reagents

AP (HY-N1201) and ZEN (HY-103447) were purchased from MedChemExpress (MCE, Shanghai, China). The Wnt pathway inhibitor IWR-1-endo (S7086) was obtained from Selleck.

The types and dilution concentrations of primary antibodies are listed in Table 1. HRP-conjugated anti-rabbit IgG (7074S) and HRP-conjugated anti-mouse IgG (7076S) were acquired from Cell Signaling Technology. MEM (ATCC modified) medium (PM150467) was procured from Wuhan Procell Life Science & Technology Co., Ltd. (Wuhan, China). Fetal bovine serum (FBS; A5669701) and penicillin streptomycin (15140-122) were purchased from Thermo Fisher Scientific Inc. (Shanghai, China). DNA Marker (A0422A01) was purchased from Tiangen Biotech (Beijing, China) Co., Ltd., and PageRuler™ Prestained Protein Ladder (26616) from Thermo Fisher Scientific Inc.

### 2.2. Cell Culture and Treatments

ST cells (CL-0219) were purchased from Wuhan Pricella Biotechnology Co., Ltd. (Wuhan, China), and they were a Sertoli cell line. They were cultured and passaged in MEM medium supplemented with 10% fetal bovine serum (FBS) and 1% penicillin streptomycin. All experiments were conducted when the cells reached 70–80% confluence and were maintained in an incubator at 37 °C with 5% CO_2_. To establish the injury model, ST cells were treated with 40 μM ZEN for 24 h. In the AP + ZEN treatment group, cells were pretreated with 1 μM AP for 24 h, followed by co-treatment with 40 μM ZEN for an additional 24 h.

### 2.3. Cell Viability and Lactate Dehydrogenase Release Assay

A Cell Counting Kit-8 (CCK-8, C0038), BeyoClick™ EdU-594 Cell Proliferation Detection Kit (C0078S), and Lactate Dehydrogenase (LDH) assay kit (C0017) were all purchased from Beyotime Institute of Biotechnology (Shanghai, China). Cell viability was measured using the Cell Counting Kit-8. ST cells were seeded in 96-well plates at a density of 3000 cells per well and treated with different concentrations of AP (0–10 μM) and ZEN (0–60 μM) to screen for appropriate injury and protective concentrations. After treatment, 10 μL of CCK-8 assay solution was added to each well, followed by incubation for 1 h. Absorbance was then measured at 450 nm using a microplate reader. Cell proliferation was evaluated using the EdU assay, and cytotoxicity was assessed by measuring LDH release into the culture medium according to the manufacturer’s instructions.

### 2.4. Flow Cytometry Analysis of Cell Cycle

DNA Content Quantitation Assay (Cell Cycle, CA1510) was purchased from Solarbio Science & Technology Co., Ltd. (Beijing, China). ST cells (3.5 × 10^5^ cells/mL) were seeded into 6-well culture plates as the C, ZEN, AP, and AP + ZEN groups. After treatment, cells were collected and prepared as single-cell suspensions, followed by fixation with pre-cooled 70% ethanol at 4 °C overnight. The fixed cells were then centrifuged and washed twice with 1× PBS. Subsequently, the cells were stained with 100 μL RNase A and 400 μL propidium iodide in the dark for 30 min. Finally, the samples were analyzed using a flow cytometer (BD FACS Canto II), and cell cycle distribution was evaluated with FlowJo software (version 10.8.1).

### 2.5. Flow Cytometry Analysis of Cell Apoptosis

A Cell Apoptosis Detection Kit (FA101) was purchased from TransGen Biotech (Beijing, China). ST cells (3.5 × 10^5^ cells/mL) were seeded into 6-well culture plates as the C, ZEN, AP, and AP + ZEN groups. After 24 h of treatment, cells were collected, washed twice with PBS, and stained with Annexin V-FITC and propidium iodide (PI) according to the manufacturer’s instructions. Cells stained separately with either Annexin V-FITC or PI were used as compensation controls. For each sample, at least 10,000 single-cell events were acquired. Based on the fluorescence intensity of Annexin V and PI, cell populations were classified as viable cells (Annexin V^−^/PI^−^), early apoptotic cells (Annexin V^+^/PI^−^), and late apoptotic cells (Annexin V^+^/PI^+^). Apoptosis rates were analyzed using a flow cytometer (BD FACS Canto II) and quantified with FlowJo software.

### 2.6. Measurement of SOD Activity, MDA Content, and T-AOC in Cells

Cells from the C, ZEN, and AP + ZEN groups were collected and centrifuged, after which the supernatant was discarded. An appropriate extraction buffer was added to the cell pellets, and the cells were disrupted via ultrasonication. To measure superoxide dismutase (SOD) activity and malondialdehyde (MDA) content, the resulting cell lysate was centrifuged at 8000 rpm and 4 °C for 10 min. The supernatant was collected and kept on ice for subsequent analysis. SOD activity and MDA content were determined using the respective assay kits (Solarbio, BC0170 for SOD; BC0020 for MDA) according to the manufacturer’s instructions. Absorbance for SOD activity was measured at 560 nm, while MDA content was measured at 532 nm and 600 nm.

For total antioxidant capacity (T-AOC) assessment, a separate aliquot of the cell lysate supernatant was centrifuged at 10000 rpm and 4 °C for 10 min. The resulting supernatant was placed on ice and analyzed using the T-AOC assay kit (FRAP; Solarbio, BC1310). Absorbance was measured at 593 nm following the kit protocol.

### 2.7. Western Blot Analysis

After processing the cells as described in Section 2.3, ST cells were digested with trypsin for 2 min. The digestion was terminated by adding MEM medium containing 10% FBS. The cell suspension was centrifuged at 1200 r/min for 4 min to collect the cell pellets. The supernatant was discarded, and 50 μL of RIPA lysis buffer (Beyotime, P0013), containing 1:100 PMSF, was added to each sample. The cells were lysed on ice for 30 min to extract total protein. After centrifuging the lysate, the supernatant was collected, and the protein concentration was determined using a BCA protein assay kit (Beyotime, P0010). Subsequently, 5× protein loading buffer (Beyotime, P0285) was added, and the samples were heated at 98 °C for 10 min for denaturation.

A total of 20 μg protein per sample was loaded and separated via SDS-PAGE using a 12.5% fast-prep PAGE gel kit (Epizyme, PG113, Shanghai, China) at 90 V for the stacking gel and 170 V for the separating gel. The separated proteins were then transferred onto a PVDF membrane (Cwbio, CW0059S, Beijing, China). The membrane was blocked with rapid blocking buffer (Epizyme, PS108P) at room temperature for 2 h. According to the molecular weight of the target proteins, the PVDF membrane was cut into strips and incubated with corresponding primary antibodies at 4 °C overnight. After washing with TBST (Epizyme, PS103S), the membrane was incubated with horseradish peroxidase (HRP)-conjugated secondary antibodies (1:1000 dilution) at room temperature for 50 min. Finally, protein bands were visualized using SuperSignal™ West Pico PLUS Chemiluminescent Substrate (Thermo, 34577) and captured with a chemiluminescence imaging system. The relative expression of target proteins was quantified by normalizing their band gray values to those of β-actin, measured using ImageJ (version 1.54).

### 2.8. RNA Extraction and RT-PCR and qRT-PCR

To identify ST cells, the expression of the specific marker genes (WT1, SOX9, and GATA4) was analyzed via reverse transcription-polymerase chain reaction (RT-PCR), which was performed in a 10 μL mixture containing 1 μL cDNA template, 0.5 μL forward primer, 0.5 μL reverse primer, 5 μL 2 × Phanta UniFi Master Mix (Vazyme, P526-02-AA, Nanjing, China), and 3 μL RNase-free water. The amplification protocol was as follows: initial denaturation at 98 °C for 30 s, followed by 30 cycles of denaturation at 98 °C for 10 s, annealing at 57 °C for 5 s, and extension at 72 °C for 10 s, as well as separation via electrophoresis using 1% agarose gel (Solarbio, A8201).

After treatment, total RNA was extracted from ST cells in each group using Trizol reagent (Vazyme, 7E0191J4). RNA concentration and purity were assessed by measuring the absorbance at 260/280 nm. Subsequently, cDNA was synthesized from RNA using the PrimeScript™ RT Master Mix (TaKaRa, RR036A, Beijing, China). Quantitative real-time PCR (qRT-PCR) was performed using Taq Pro Universal SYBR qPCR Master Mix (Vazyme, Q712-03) to analyze the expression levels of the target genes. The qRT-PCR was performed in a 20 μL mixture containing 1 μL cDNA template, 0.5 μL forward primer, 0.5 μL reverse primer, 10 μL SYBR qPCR Mix, and 8 μL RNase-free water. The amplification protocol was as follows: initial denaturation at 95 °C for 30 s, followed by 40 cycles of denaturation at 95 °C for 10 s and annealing/extension at 60 °C for 30 s. The relative expression of target genes was normalized to the average of three internal reference genes and calculated using the 2^−ΔΔCT^ method [35].

The primer sequences used in this study were designed with NCBI Primer-BLAST (https://www.ncbi.nlm.nih.gov/tools/primer-blast/, accessed on 23 December 2025). The primer sequences are listed in Table 2 and GAPDH was used as the internal control.

### 2.9. RNA-Seq and Enrichment Analysis

To further investigate the molecular mechanism by which AP alleviates ZEN-induced cellular injury, transcriptome sequencing analysis was performed, and the test samples were divided into four groups: C group, ZEN group, ZEN + AP group, and AP group. Each group was provided with 6 technical replicates of samples. The concentration and purity of the extracted total RNA were measured using a Nanodrop 2000 (Thermo Fisher Scientific, Shanghai, China), while RNA integrity was assessed via agarose gel electrophoresis. The RNA quality number (RQN) was determined using an Agilent 5300 system (Agilent Technologies, Shanghai, China). The RNA samples used for library construction met the following criteria: total amount ≥ 1 μg, concentration ≥ 30 ng/μL, RQN > 6.5, and an OD260/280 ratio between 1.8 and 2.2. Differentially expressed genes (DEGs) were identified with a *p*-value < 0.05 and |FC| > 1.2. These DEGs were subjected to Gene Ontology (GO) functional enrichment analysis and Kyoto Encyclopedia of Genes and Genomes (KEGG) pathway enrichment analysis. Data analysis and visualization were conducted on the NovaSeq X Plus platform.

### 2.10. Statistical Analysis

All experiments were independently repeated at least three times. Data were analyzed using Student’s *t*-test for comparisons between two groups with GraphPad Prism software (version 10.1.2). *p* < 0.05 was considered statistically significant.

## 3. Results

### 3.1. Effects of Different Concentrations of AP and ZEN on ST Cell Viability

**Figure 1:**
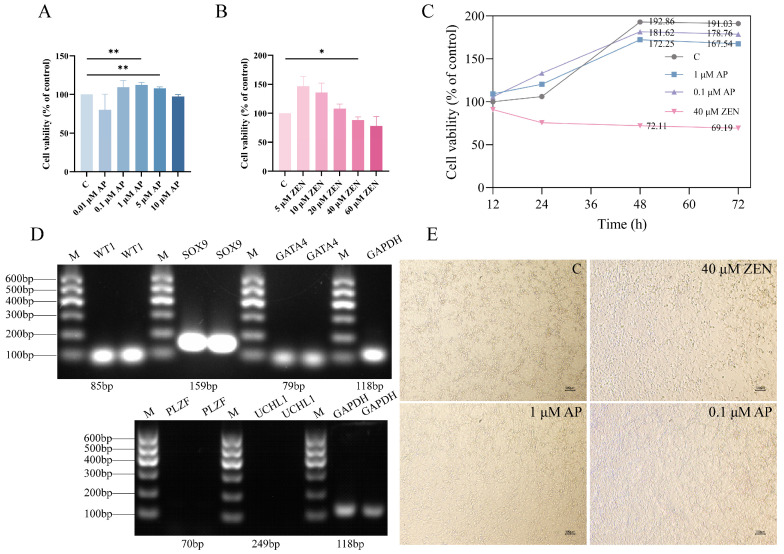
Effects of apigenin (AP) and zearalenone (ZEN) on Swine Testis (ST) cell viability. (**A**) Viability of ST cells treated with AP for 24 h. (**B**) Viability of ST cells treated with ZEN for 24 h. (**C**) Proliferation curves of ST cells determined through continuous monitoring with the CCK-8 assay. (**D**) RT-PCR analysis confirmed the expression of ST cell markers (WT1, SOX9, GATA4) and no expression of germ cell markers PLZF and UCHL1 in ST cells. (**E**) Bright-field images of cells treated with AP or ZEN for 48 h. Scale bar, 100 μm. Data are presented as mean ± SEM from more than three independent experiments. * *p* < 0.05, ** *p* < 0.01.

To evaluate the effect of AP on ST cell viability, cells were exposed to varying concentrations of AP for 24 h. The results show that AP at concentrations ranging from 0.1 to 5 μM promoted ST cell proliferation, with the 1 μM and 5 μM AP treatment groups exhibiting significantly enhanced proliferation rates compared to the control (C) group (Figure 1A, *p* < 0.01). Based on these findings, treatment with 1 μM AP for 24 h was selected as the optimal condition for the protective intervention model.

Subsequently, to determine the appropriate injury-inducing concentration of ZEN, ST cells were exposed to different concentrations of ZEN for 24 h. The results indicate that cell proliferation was progressively inhibited with increasing ZEN concentration. Specifically, 40 μM ZEN significantly reduced the cell proliferation rate (*p* < 0.05) and was therefore selected as the injury-inducing concentration for establishing the cellular injury model (Figure 1B).

We monitored the temporal effects of AP and ZEN on cellular growth and generated the corresponding cell proliferation curves (Figure 1C). As shown in Figure 1D, RT-PCR analysis confirmed the expression of ST cell-specific marker genes (WT1, SOX9, and GATA4) in the ST cells, validating their cellular identity; in contrast, no expression of germ cell markers PLZF and UCHL1 was observed in ST cells. Representative bright-field microscopic images of cells following 48 h of treatment are presented in Figure 1E. The results show that cells treated with 1 μM and 0.1 μM AP exhibited robust growth and maintained healthy morphology. In contrast, treatment with 40 μM ZEN resulted in extensive cell death.

### 3.2. Protective Effect of AP on ZEN-Induced Decrease in ST Cell Viability

**Figure 2:**
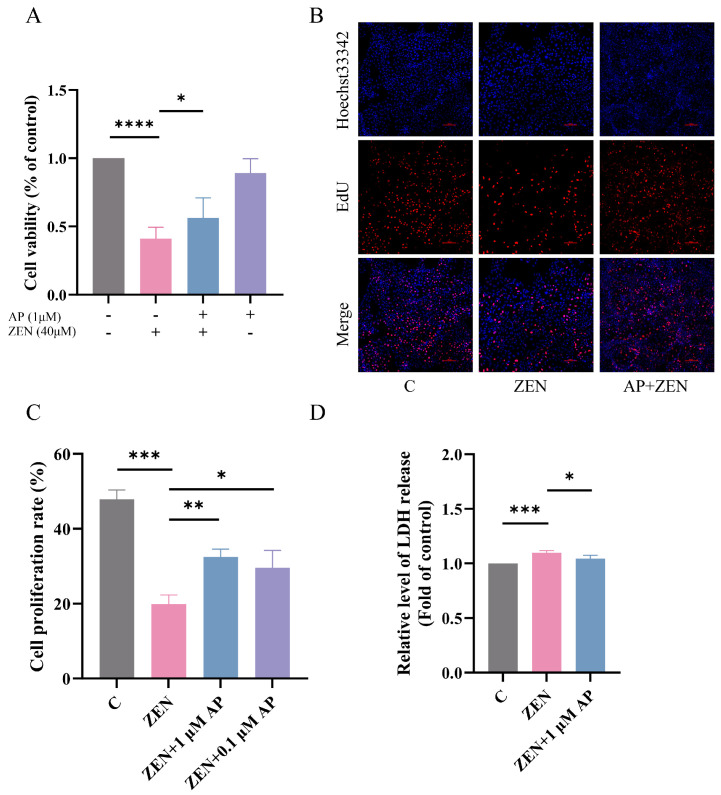
Protective effect of AP against ZEN-induced decrease in ST cell proliferation. (**A**) Effect of combined AP and ZEN treatment on ST cell viability measured via CCK-8 assay. (**B**,**C**) Effect of combined AP and ZEN treatment on ST cell proliferation detected via EdU assay. Scale bar, 100 μm. (**D**) Effect of combined AP and ZEN treatment on lactate dehydrogenase (LDH) release in ST cells. For the protective model, cells were pretreated with 1 μM or 0.1 μM AP for 24 h, followed by continued treatment with 40 μM ZEN for the same duration. Data are presented as mean ± SEM from more than three independent experiments. * *p* < 0.05, ** *p* < 0.01, *** *p* < 0.001, **** *p* < 0.0001.

Based on preliminary experimental results, 40 μM ZEN was employed to establish the ST cell injury model, and 1 μM AP was selected as the intervention condition. In the AP + ZEN treatment group, cells were pretreated with 1 μM AP for 24 h, followed by co-treatment with 40 μM ZEN for an additional 24 h. Cell viability was assessed using the CCK-8 assay, and the results show that 1 μM AP significantly attenuated the ZEN-induced decrease in ST cell viability (Figure 2A). Further validation using the 5-ethynyl-2′-deoxyuridine (EdU) cell proliferation assay confirmed that 1 μM AP significantly mitigated the ZEN-induced reduction in ST cell proliferation (Figure 2B,C). Lactate dehydrogenase (LDH) serves as a common cytotoxicity biomarker that is primarily localized intracellularly under physiological conditions but is released into the extracellular space upon cell membrane damage. Measuring LDH release in the culture supernatant revealed that 1 μM AP significantly reversed the ZEN-induced elevation in LDH release (Figure 2D), indicating that AP alleviates ZEN-induced cytotoxicity.

### 3.3. The Addition of AP Reversed the Effect of ZEN on the Cell Cycle Distribution of ST Cells

**Figure 3:**
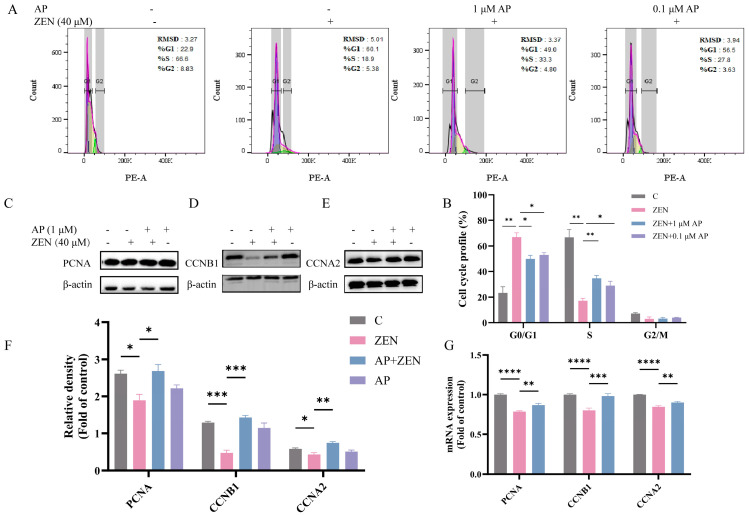
Identification of the alleviation effects of AP on ZEN-induced cell cycle arrest in ST cells. (**A**,**B**) Cell cycle analysis via flow cytometry. (**C**–**F**) Protein expression levels of PCNA, CCNB1, and CCNA2 detected via Western blot. β-actin was used as the internal control. (**G**) mRNA expression levels of PCNA, CCNB1, and CCNA2 measured via qRT-PCR. Data are presented as mean ± SEM from three independent experiments (*n* = 3). * *p* < 0.05, ** *p* < 0.01, *** *p* < 0.001, **** *p* < 0.0001.

To further elucidate the mechanism underlying ZEN-induced suppression of ST cell viability, the cell cycle distribution was analyzed using flow cytometry. The results showed that, compared with the control (C) group, the ZEN treatment group exhibited a significant increase in the proportion of cells in G1 phase and a concomitant significant decrease in S phase cells. In contrast, AP intervention reversed the ZEN-induced G1 phase arrest and restored the proportion of cells in S phase (Figure 3A,B).

Subsequently, alterations in the expression of proliferation-related proteins were analyzed via Western blot. The results show that ZEN treatment significantly reduced the protein levels of proliferating cell nuclear antigen (PCNA), cyclin B1 (CCNB1), and cyclin A2 (CCNA2) compared with the control group, whereas AP intervention restored their expression levels (Figure 3C–F). A consistent trend was observed at the transcriptional level: ZEN significantly downregulated levels of the mRNA for PCNA, CCNB1, and CCNA2. After the addition of AP, these gene levels were restored in ST cells (Figure 3G).

Collectively, these findings indicate that ZEN induces cell cycle arrest at the G1 phase and suppresses S phase progression, which may subsequently lead to ST cell apoptosis. In contrast, AP treatment effectively ameliorates ZEN-induced cell cycle dysregulation and aberrant expression of the relevant molecules.

### 3.4. AP Alleviates ZEN-Induced Apoptosis in ST Cells

**Figure 4:**
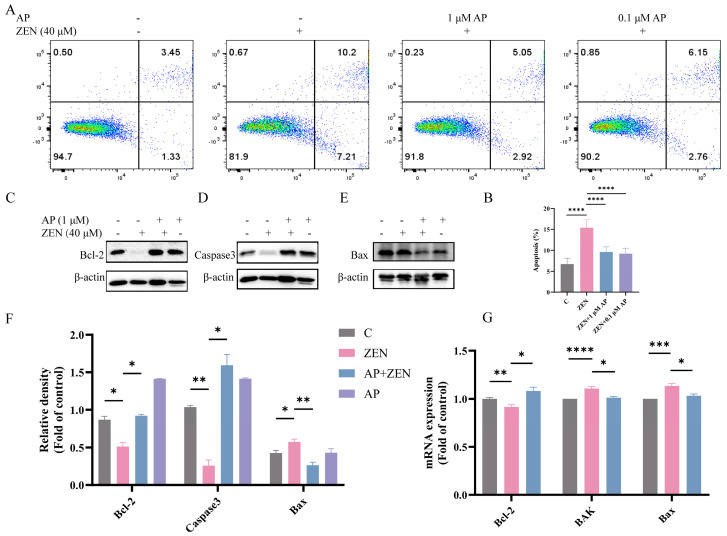
Identification of the alleviation effects of AP on ZEN-induced apoptosis in ST cells. (**A**,**B**) Apoptosis detection via flow cytometry. (**C**–**F**) Protein expression levels of Bcl-2, Caspase3, and Bax detected via Western blot. β-actin was used as a loading control. (**G**) mRNA expression levels of Bcl-2, BAK, and Bax measured via qRT-PCR. Data are presented as mean ± SEM from three independent experiments (*n* = 3). * *p* < 0.05, ** *p* < 0.01, *** *p* < 0.001, **** *p* < 0.0001.

To determine whether ZEN induces apoptosis in ST cells, Annexin V-FITC/PI dual staining combined with flow cytometric analysis was performed. The results show that ZEN treatment significantly elevated the apoptosis rate of ST cells, with apoptotic cells distributed in both the Annexin V^+^/PI^+^ (late apoptosis) and Annexin V^+^/PI^−^ (early apoptosis) quadrants. In contrast, AP intervention significantly reduced the proportion of ZEN-induced apoptotic cells (Figure 4A,B).

Further Western blot analysis revealed that ZEN treatment decreased the expression levels of the anti-apoptotic proteins B-cell lymphoma-2 (Bcl-2) and Caspase-3, while concomitantly increasing the expression of the pro-apoptotic protein Bcl-2-associated X protein (Bax), which represents typical hallmarks of apoptotic progression. AP intervention significantly reversed these alterations in protein expression (Figure 4C–F). At the transcriptional level, ZEN significantly downregulated Bcl-2 mRNA expression and upregulated the expression of BAK and Bax. After the addition of AP, these gene levels were restored in ST cells (Figure 4G).

Collectively, these results show that AP effectively ameliorates ZEN-induced apoptosis in ST cells and modulates the expression of apoptosis-related molecules at both the protein and transcriptional levels.

### 3.5. AP Alleviates ZEN-Induced Oxidative Stress and Inflammation in ST Cells

**Figure 5:**
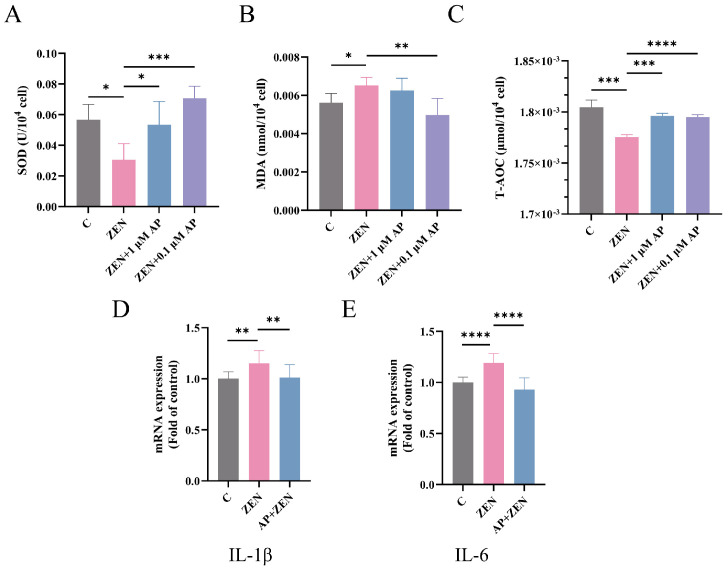
Detection of the expression levels of SOD (**A**), MDA (**B**), and T-AOC (**C**) in ST cells after different treatments. (**D**,**E**) mRNA levels of IL-1β and IL-6 in ST cells measured via qRT-PCR. Data are presented as mean ± SEM from three independent experiments (*n* = 3). * *p* < 0.05, ** *p* < 0.01, *** *p* < 0.001, **** *p* < 0.0001.

As shown in Figure 5A,C, compared with the control (C) group, ZEN treatment significantly decreased superoxide dismutase (SOD) activity (*p* < 0.05) and total antioxidant capacity (T-AOC) level (*p* < 0.001) in ST cells. In contrast, supplementation with 1 μM AP significantly increased SOD activity (*p* < 0.05) and T-AOC level (*p* < 0.001), while 0.1 μM AP also markedly enhanced SOD activity (*p* < 0.001) and T-AOC level (*p* < 0.001). Conversely, the malondialdehyde (MDA) content in the ST cells of the ZEN treatment group was significantly elevated (*p* < 0.05), whereas intervention with 0.1 μM AP effectively suppressed MDA accumulation (*p* < 0.01) (Figure 5B).

Regarding inflammation-related genes, ZEN significantly upregulated the levels of the mRNA for interleukin-1β (IL-1β) and interleukin-6 (IL-6), whereas AP supplementation restored their expression to near-baseline levels (Figure 5D,E).

Collectively, these findings demonstrate that AP effectively alleviates ZEN-induced oxidative stress and inflammatory responses in ST cells, exhibiting significant restorative effects across multiple antioxidant and pro-inflammatory biomarkers.

### 3.6. RNA-Seq Analysis for the Underlying Molecular Mechanisms of the AP-Reversed Effect of ZEN in ST Cells

**Figure 6:**
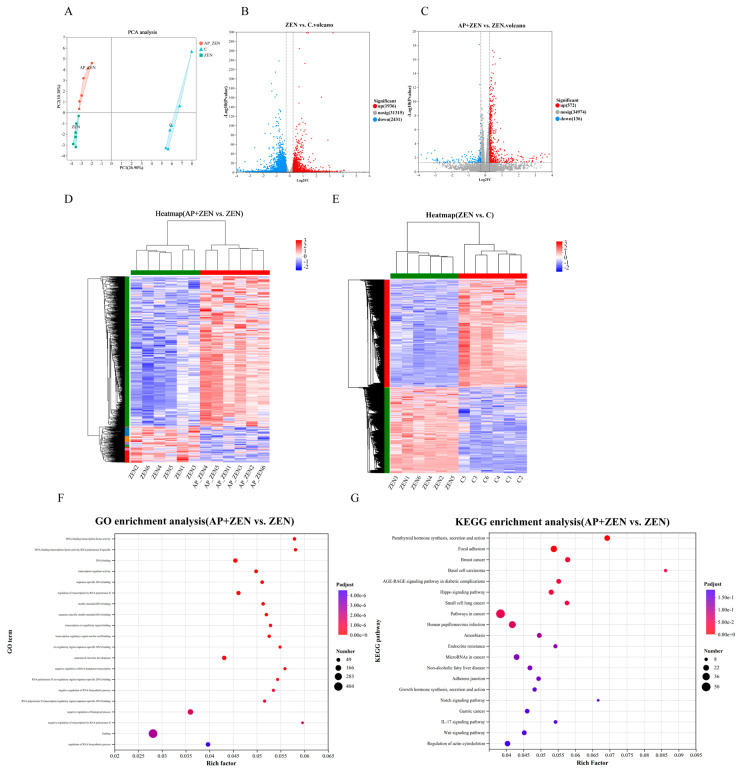
Analysis of differentially expressed genes of ZEN-treated and AP + ZEN-treated ST cells from RNA-Seq assays. (**A**) PCA plot and (**D**,**E**) cluster heatmap demonstrate significant differences among the C, ZEN, and AP + ZEN groups. (**B**,**C**) Volcano plots display the number of DEGs in the ZEN vs. C comparison and the AP + ZEN vs. ZEN comparison. (**F**,**G**) GO and KEGG enrichment analysis between the AP + ZEN and ZEN groups.

To investigate the molecular mechanism by which AP alleviates ZEN-induced apoptosis in ST cells, transcriptome sequencing was performed. Principal component analysis showed clear separation among the C, ZEN, and AP + ZEN groups, indicating significant differences in gene expression profiles (Figure 6A). Using the threshold of |FC| > 1.2 and *p* < 0.05, 4367 differentially expressed genes (DEGs) were identified in the ZEN-treated group vs. the C group, including 1936 up-regulated and 2431 down-regulated genes. In the AP + ZEN vs. ZEN comparison, 708 DEGs were detected, comprising 572 up-regulated and 136 down-regulated genes (Figure 6B,C). Cluster heatmaps further confirmed transcriptomic differences among the three groups (Figure 6D,E). The RNA-Seq accession number is PRJNA1370341.

GO enrichment analysis revealed that the DEGs were significantly enriched in transcriptional regulation functions, including DNA-binding transcription factor activity, sequence-specific DNA binding, and RNA polymerase II cis-regulatory region binding. These functions are closely associated with precise regulation of gene expression and play central roles in cellular proliferation and differentiation processes (Figure 6F). Pathway annotation using the Majorbio Cloud Platform (www.majorbio.com) revealed that, compared with the ZEN group, the AP + ZEN group was significantly enriched in the Wnt signaling pathway (Figure 6G). KEGG pathway enrichment analysis revealed significant enrichment of differentially expressed genes in pathways involved in cell growth, proliferation, and differentiation, including the Wnt, Hippo and Notch signaling pathways, and so on.

**Figure 7:**
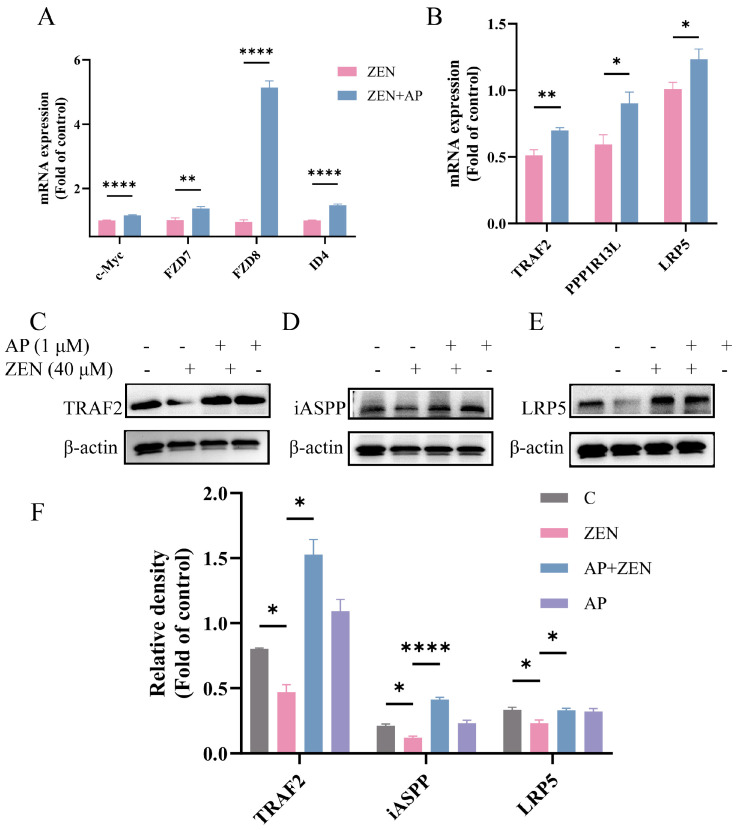
Key genes and proteins in the Wnt signaling pathway and other signaling pathways were verified via qRT-PCR and Western blot. (**A**,**B**) mRNA expression levels of c-Myc, FZD7, FZD8, ID4, TRAF2, PPP1R13L and LRP5 were significantly up-regulated after AP intervention. (**C**–**F**) ZEN-induced suppression of LRP5, iASPP, and TRAF2 protein expression was alleviated by AP. The β-actin of 3E and 7J is the same because they are derived from the same PVDF membrane. Data are presented as mean ± SEM from three independent experiments (*n* = 3). * *p* < 0.05, ** *p* < 0.01, **** *p* < 0.0001.

Notably, key genes in this pathway, such as FZD7, FZD8, TRAF2, PPP1R13L, and LRP5, were significantly up-regulated at the transcriptional level, and the expression changes in randomly selected genes were verified via qRT-PCR (Figure 7A,B). Furthermore, Western blot results confirmed that AP significantly activated Wnt pathway-related proteins. Specifically, ZEN treatment reduced LRP5 protein levels, while AP intervention reversed this trend. In addition, ZEN-induced suppression of iASPP and TRAF2 protein expression was also alleviated with AP treatment (Figure 7C–F). Taken together, both transcriptomic and protein-level data demonstrate that ZEN-induced injury to ST cells and the protective effect of AP are closely associated with the regulation of the Wnt signaling pathway.

### 3.7. The Protective Effect of AP Against ZEN-Induced ST Cell Injury Is Mediated by the Wnt Signaling Pathway

**Figure 8:**
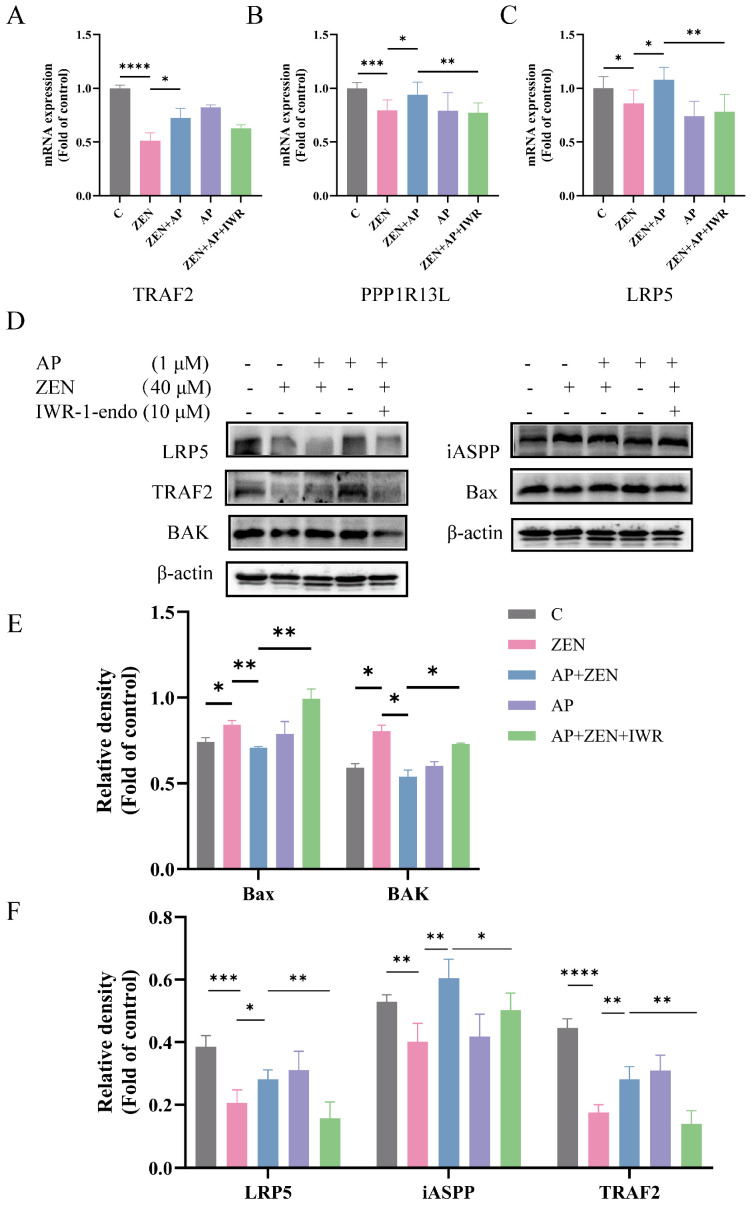
Effects of the Wnt signaling pathway inhibitor IWR-1-endo on Wnt pathway-related genes (**A**–**C**) and LRP5, iASPP, TRAF2 proteins, as well as pro-apoptotic proteins (Bax, BAK) in ST cells (**D**–**F**). Data are presented as mean ± SEM from three independent experiments (*n* = 3). * *p* < 0.05, ** *p* < 0.01, *** *p* < 0.001, **** *p* < 0.0001.

To further investigate whether AP alleviates ZEN-induced toxicity in ST cells through the Wnt signaling pathway, cells were pretreated with 10 μM Wnt pathway inhibitor IWR-1endo for 24 h. The results show that the addition of IWR-1endo significantly reduced the expression of TRAF2, iASPP, and LRP5 at both the mRNA (Figure 8A–C) and protein (Figure 8D,F) levels compared to the AP + ZEN group. These findings indicate that the protective effect of AP against ZEN-induced ST cell injury is dependent on the activation of the Wnt signaling pathway. Moreover, the coordinated expression changes in LRP5, iASPP, and TRAF2 suggest a potential functional association or indirect interaction among them, collectively contributing to the cytoprotective effects mediated by AP.

Subsequently, IWR-1-endo treatment significantly attenuated the protective effect of AP against ZEN-induced apoptosis, as evidenced by a marked increase in the expression levels of the pro-apoptotic proteins Bax and BAK (Figure 8D,E). Altogether, AP plays a key role in alleviating ZEN-induced apoptosis and oxidative stress in ST cells by upregulating LRP5 through the modulation of the Wnt signaling pathway.

## 4. Discussion

ZEN adversely affects animal growth and development, impairs reproductive capacity [9], induces oxidative stress [12] and cell death [36], and may even promote tumorigenesis [37]. ZEN mainly affects tissues, including the ovary, uterus, breast, testis and other sites with a high expression of estrogen receptors. At low doses, it may promote cell proliferation and carcinogenesis; conversely, high doses induce severe cellular damage, encompassing endoplasmic reticulum stress, oxidative stress, and DNA and mitochondrial damage, which results in cell cycle arrest and apoptosis [38,39,40]. The present study demonstrates that ZEN exhibits pronounced cytotoxicity, resulting in injury to ST cells, reduced cell viability, altered cellular morphology and structure, and induction of apoptosis. These findings further substantiate the reproductive toxicity of ZEN. Conversely, AP is a common dietary flavonoid possessing antioxidant, anti-inflammatory, and other biological properties, rendering it a potential candidate for counteracting germ cell apoptosis [27,28,30,41,42,43]. Therefore, we selected ST cells as an in vitro model to investigate the protective effects of AP against ZEN-induced injury and elucidate its underlying mechanisms.

We first investigated the effects of ZEN and AP on ST cell proliferation. Previous studies have demonstrated that ZEN significantly inhibits the proliferative activity of germ cells [12] and intestinal cells [44]. Consistent with these findings, our study revealed that ZEN concentrations at or above 40 μM significantly reduced ST cell viability. Conversely, AP at concentrations ranging from 0.1 to 5 μM significantly enhanced ST cell viability. Additionally, AP has been reported to inhibit spermatogonial proliferation by downregulating the Prmt7/Akt pathway, thereby modulating male reproductive health [41]. AP has also demonstrated beneficial effects in protecting against reproductive toxicity, including alleviating acrylonitrile-induced inflammation and apoptosis in testicular cells [32] and conferring protection against subchronic sperm injury [33]. These findings collectively suggest that AP may possess a favorable biosafety profile for the reproductive system. Therefore, the present study selected AP as a potential protective agent to investigate its interventional effects on ZEN-induced toxicity in ST cells. As anticipated, AP significantly ameliorated the ZEN-induced decline in cell viability. Furthermore, ZEN treatment significantly increased LDH release into the cell culture supernatant, which is indicative of ZEN-induced cellular injury. In contrast, AP supplementation effectively suppressed the ZEN-induced elevation in LDH release, further corroborating its protective effects against ZEN toxicity.

The cell cycle serves as an important functional indicator for assessing cellular metabolism and physiological and pathological states, and its dysregulation is intimately associated with cell proliferative activity [45]. Previous investigations have demonstrated that ZEN can disrupt normal cell cycle progression in various cell types [46,47,48]. The results of the present study demonstrate that ZEN treatment significantly reduced the proportion of ST cells in the S phase while concomitantly increasing the proportion in the G1 phase. The S phase represents a critical stage for DNA synthesis, and impaired or erroneous DNA replication can inhibit cell division or induce mutations, thereby leading to the generation of abnormal cells or developmental malformations [49]. A decrease in the proportion of S-phase cells indicates diminished DNA synthesis capacity and a potential increase in abnormal cell populations, which is consistent with the previously observed decline in cell viability. In cell cycle regulation, PCNA, CCNA2, and CCNB1 participate in DNA repair, replication, and cycle control, with their expression peaking during the S phase [50]. As demonstrated in our results, the mRNA expression levels of PCNA, CCNA2, and CCNB1 were significantly reduced in ST cells following ZEN treatment. Notably, the increase in G1-phase ST cells following ZEN exposure implies the occurrence of cell death (Figure 3).

We evaluated the effect of ZEN on ST cell apoptosis using Annexin V-FITC/PI staining, and the results show that ZEN treatment significantly increased the apoptosis rate (Figure 4). Although numerous studies have reported that ZEN can induce apoptosis in germ cells, evidence in ST cells remains limited. Bcl-2 and Bax are pivotal regulatory proteins in apoptosis: Bcl-2 exerts an anti-apoptotic function, whereas Bax promotes the apoptotic cascade [51]. A decrease in Bcl-2 expression coupled with an increase in Bax expression significantly enhances mitochondrial membrane permeability, thereby facilitating the release of cytochrome C into the cytoplasm [52]. The accumulated cytochrome C in the cytoplasm activates Caspase-3—a member of the cysteine aspartic acid protease family and one of the central executioners of apoptosis [53]. Activated Caspase-3 undergoes proteolytic cleavage into smaller fragments, accompanied by a corresponding decrease in its full-length form, ultimately driving the cell toward apoptotic death [54]. Our results show that ZEN-induced apoptosis in ST cells significantly promoted Bax expression and suppressed the expression of both Bcl-2 and the full-length form of Caspase-3.

In evaluating oxidative stress status, the present study measured SOD, MDA, and T-AOC as key indicators. SOD plays a critical role in maintaining the oxidative/antioxidant equilibrium in cells, while MDA, as a principal product of lipid peroxidation, reflects the magnitude of oxidative stress [12]. Studies have demonstrated that AP can alleviate acrylonitrile-induced lipid peroxidation and exert protective effects on testicular cells [33]. The present study found that ZEN treatment significantly increased MDA content, while SOD activity and T-AOC levels were markedly reduced, indicating that ZEN induced substantial oxidative stress. In contrast, AP intervention notably reversed these alterations, suggesting that AP enhances the concentration of intracellular antioxidants and reduces the excessive accumulation of free radicals induced by ZEN. Therefore, AP supplementation can enhance the antioxidant capacity of ST cells.

The apoptosis-stimulating protein of p53 (iASPP) is an inhibitor of p53 and a key anti-apoptotic protein. Studies have demonstrated that iASPP interacts with p53 to inhibit p53-mediated apoptosis and interferes with the transcriptional activity of downstream target genes, thereby modulating cell proliferation and survival [55]. This effect results in increased Bcl-2 expression and decreased Bax expression. The inhibitor of DNA binding 4 (ID4) is a highly conserved mammalian DNA-binding inhibitory protein involved in the regulation of cell differentiation and proliferation [56].

Notably, the Wnt/β-catenin signaling pathway regulates numerous fundamental processes essential for embryonic development, adult tissue homeostasis, and cancer progression [57]. Wnt agonists have been observed to promote hair follicle growth [58], and activated Wnt signaling enhances the proliferation of human dermal papilla cells [59]. Frizzled 7 and 8 (FZD7 and FZD8) are key receptors in the Wnt/β-catenin signaling pathway, playing crucial roles in maintaining stem cell properties, promoting cell proliferation, and enhancing cell migration through the activation of canonical Wnt signaling [60]. Low-density lipoprotein receptor-related protein 5 (LRP5) functions as a co-receptor in this pathway, forming a receptor complex with FZD7/8. The phosphorylation of its intracellular domain recruits Axin to the plasma membrane, thereby inhibiting the phosphorylation and degradation of β-catenin. This promotes the cytoplasmic accumulation of β-catenin and its subsequent nuclear translocation, where it initiates the transcription of downstream target genes such as c-Myc, ultimately activating the Wnt/β-catenin pathway to promote cell proliferation [61].

Our results show that ZEN treatment significantly reduced the expression of proliferation-related proteins, including tumor necrosis factor receptor-associated factor 2 (TRAF2), iASPP, and LRP5 in ST cells, whereas AP intervention reversed this trend and activated the Wnt signaling pathway. Importantly, when the Wnt pathway inhibitor IWR-1-endo was administered, the regulatory effect of AP on the Wnt pathway was attenuated, and its protective effects on ST cells were significantly diminished. In conclusion, the present study not only elucidates the mechanisms underlying ZEN-induced toxicity in ST cells but also reveals the molecular basis by which AP ameliorates ZEN-induced cellular injury through the modulation of the Wnt and other associated signaling pathways.

**Figure 9:**
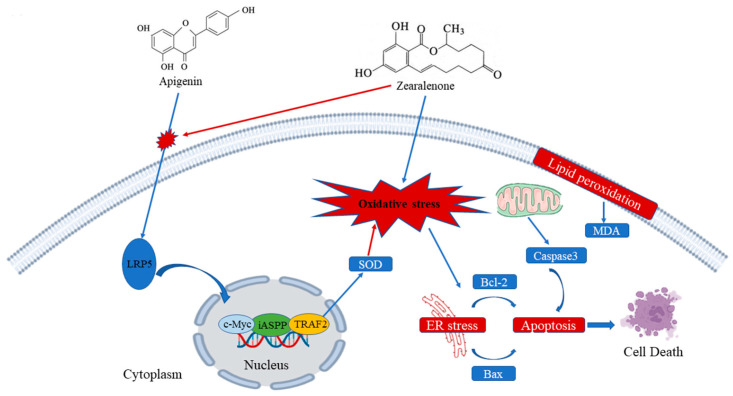
Mechanism of AP in alleviating ZEN-induced cytotoxicity in ST cells. Arrows: promotion.

ZEN induces oxidative stress, cell apoptosis, and disrupts cell cycle progression in ST cells. Mechanistically, ZEN suppresses the expression of LRP5, thereby inhibiting the canonical Wnt/β-catenin signaling pathway and its downstream targets. AP counteracts these toxic effects by upregulating LRP5 expression, which activates the Wnt signaling pathway. This activation promotes cell proliferation and inhibits apoptosis. The essential role of the Wnt pathway in AP-mediated protection was confirmed, as its specific inhibitor (IWR-1-endo) abrogated the beneficial effects. Furthermore, AP enhances the expression of key anti-apoptotic proteins iASPP and TRAF2. Collectively, this study elucidates that AP alleviates ZEN-induced reproductive toxicity primarily through the activation of the LRP5/Wnt signaling pathway (Figure 9).

## 5. Conclusions

This study confirms that ZEN significantly inhibits ST cell viability, reduces the proportion of S-phase cells, and induces apoptosis and oxidative stress in ST cells. In contrast, AP intervention effectively alleviates ZEN-induced injury. More importantly, we revealed the potential of AP in reproductive protection and elucidated its mechanism of action: by activating the Wnt signaling pathway and upregulating the expression of the co-receptor LRP5, AP antagonizes ZEN-induced ST cell injury. This study not only further clarifies the adverse effects of ZEN on male reproduction but also provides a theoretical basis for the potential use of AP as a feed additive to prevent mycotoxicosis in pigs.

## Figures and Tables

**Table 1:** Antibody information.

Antibody Name	Catalog No.	Vendor	Dilution Factor
PCNA	2586S	Cell Signaling Technology	1:2000
CCNB1	12231S	Cell Signaling Technology	1:1000
CCNA2	4656S	Cell Signaling Technology	1:2000
Bcl-2	F0125	Selleck	1:1000
Caspase3	14220S	Cell Signaling Technology	1:1000
BAX	50599-2-Ig	Proteintech	1:20,000
TRAF2	26846-1-AP	Proteintech	1:1000
iASPP	18590-1-AP	Proteintech	1:1000
LRP5	24899-1-AP	Proteintech	1:1000
β-actin	1115-1-RR	Proteintech	1:10,000

**Table 2:** RNA primer sequences.

Gene	Primer Sequence (5′-3′)	Accession No	Product/bp	The Annealing Temperature (°C)
*Bcl-2*	Forward: GGATAACGGAGGCTGGGATG	NC_010443.5	147	59.96
	Reverse: TTATGGCCCAGATAGGCACC			59.23
*BAK*	Forward: ATCAACCGGCGATACGACTC	NC_010449.5	155	59.97
	Reverse: TAGCCAAAGCCCAGAAGAGC			60.04
*Bax*	Forward: CAGCTCTGAGCAGATCATGAAG	NC_010448.4	72	58.87
	Reverse: ATTCGCCCTGCTCGATCCT			61.14
*CCNB1*	Forward: AGGGCTTACAAAGCACATGACTA	NC_010458.4	78	59.99
	Reverse: AGCTGGGCTAGAGTGCTGAT			60.69
*CCNA2*	Forward: CTAACATTGCAGCAGACGGC	NC_010450.4	174	59.9
	Reverse: CTTAAGAGGCGCAACCCGT			60.38
*c-Myc*	Forward: AAAAGGTCGGAATCGGGGTC	NC_010446.5	160	60.04
	Reverse: CCAACTTAGCCCTCTTGGCA			59.96
*FZD7*	Forward: TGAGGCGCTCATGAACAAGT	NC_010457.5	163	59.96
	Reverse: CATGTAGGGCGCTGTAGGAT			59.32
*FZD8*	Forward: CACCTACATGCCCAACCAGT	NC_010452.4	167	59.96
	Reverse: GAGGCAGGGGCTTCTTGTAG			60.11
*GATA4*	Forward: CTTGCAATGCGGAAAGAGGG	NC_010456.5	79	59.83
	Reverse: GACCTGCTGACGTCTTCGAT			59.83
*GAPDH*	Forward: GTCGGTTGTGGATCTGACCTGC	NC_010447.5	118	63.42
	Reverse: GTCCTCAGTGTAGCCCAGGATG			62.12
*IL-1β*	Forward: TCTCCTCTTTACGCAGGTTTCT	NC_010445.4	126	59.1
	Reverse: ATCTCTTTGGGGCCATCAGC			60.11
*IL-6*	Forward: CTGGGTTCAATCAGGAGACCT	NC_010451.4	165	59.09
	Reverse: TTCCCTTTTGCCTCAGGGTC			59.89
*ID4*	Forward: CAAGCAGGGCGACAGCATTC	NC_010449.5	130	62.26
	Reverse: CTTTCCTCCGGTGGCTTTTTC			59.73
*LRP5*	Forward: AATCATGCTCTTCCGACCCTC	NC_010444.4	145	59.86
	Reverse: GGAGAACTGGAAGTCCACCG			60.04
*PLZF*	Forward: CGCAAGGCTCGGTATCTCAA	NC_010451.4	70	60.18
	Reverse: CACTGGCATACCCACTCTCC			59.82
*PCNA*	Forward: CGTGAACCTCACCAGCATGT	NC_010459.5	243	60.6
	Reverse: TCTCGGCATATACGTGCAAAT			57.89
*PPP1R13L*	Forward: AAGTCCCAAGGTGCTCAAGG	NC_010448.4	93	59.89
	Reverse: CCGGGAACAGGTTAGACGAC			60.11
*SOX9*	Forward: CGGAGCTCAGCAAGACTCTG	NC_010454.4	159	60.46
	Reverse: GGCCGTTCTTCACCGACTTT			60.88
*TRAF2*	Forward: ATCGAAGCCCTGAGCAACAA	NW_018084833.1	130	59.96
	Reverse: TCCAGATGAAGACGCCATCG			59.9
*UCHL1*	Forward: CAGTAGCCAATAATCAGGAC	NC_010450.4	249	53.18
	Reverse: AAGGCATCCGACCATCAAG			57.52
*WT1*	Forward: TGTCAGCGAAAGTTCTCCCG	NC_010444.4	85	60.32
	Reverse: AGCTGAAGGGCTTTTCACTTG			59.04

## Data Availability

The original contributions presented in this study are included in the article. Further inquiries can be directed to the corresponding authors.
